# Construction of an Evaluation System and Comprehensive Assessment of the Suitability of Different Processing Peppers for Mechanized Transplanting and Harvesting

**DOI:** 10.3390/plants15101441

**Published:** 2026-05-08

**Authors:** Biyi Liu, Shudong Zhou, Sha Yang, Jie Li, Wei Peng, Zhixuan Wang, Jingxuan Kuang, Junwei Wang

**Affiliations:** 1 College of Horticulture, Hunan Agricultural University, Changsha 410128, China; 2 Institute of Vegetables, Hunan Academy of Agricultural Sciences, Changsha 410125, China; 3 Key Laboratory for Vegetable Biology of Hunan Province, Engineering Research Center for Horticultural Crop Germplasm Creation and New Variety Breeding, Ministry of Education, Changsha 410128, China; 4 Key Laboratory for Evaluation and Utilization of Gene Resources of Horticultural Crops (Vegetables, Tea, etc.), Ministry of Agriculture and Rural Affairs of China, Changsha 410128, China; 5 Yuelushan Laboratory, Changsha 410128, China

**Keywords:** processing pepper, mechanized operation, biomechanical properties, multi-criteria decision-making, CRITIC–VIKOR model

## Abstract

To address the current mismatch between processing pepper cultivars and the requirements of mechanized production, this study aims to construct a comprehensive evaluation model for the suitability of mechanized transplanting and harvesting, thereby screening highly adaptable varieties. An evaluation system comprising eight indicators for the transplanting stage and thirteen indicators for the harvesting stage was established using 105 processing pepper varieties (including 56 erect-fruit and 49 pendent-fruit peppers). Variation analysis, hierarchical clustering, principal component analysis (PCA), and Pearson correlation analysis were integrated to reveal the clustering effects of the cultivars and the synergistic and antagonistic relationships among the indicators. Furthermore, a combined CRITIC–VIKOR model was applied to conduct a multi-criteria comprehensive ranking of mechanization suitability. The results indicated that the biomechanical properties of processing peppers exhibited a significantly higher degree of variation than conventional morphological indicators (e.g., the coefficient of variation for lodging resistance reached 93.60%). Significant differences were observed in the mechanization adaptation mechanisms between the two pepper types: erect-fruit peppers were primarily limited by fruiting branch toughness (weight: 5.907%), whereas pendent-fruit peppers were mainly constrained by fruit morphological uniformity. Compared with the traditional PCA model, the CRITIC–VIKOR model effectively identified varieties with critical biomechanical defects by constraining the “individual regret value”, which highly aligns with Liebig’s Law of the Minimum in mechanized operations. Based on this model, varieties with superior comprehensive mechanization adaptability were successfully identified, notably C21, C55, and C23 (erect-fruit peppers), and D20, D11, and D19 (pendent-fruit peppers). This study provides a theoretical foundation and mathematical modeling support for the directional breeding of mechanization-suitable cultivars, the integration of agronomy and agricultural machinery, and the quantitative evaluation of multi-trait pyramiding in processing peppers.

## 1. Introduction

As a global cash crop and spice, pepper plays a significant role in the global agricultural economy [1,2]. China is among the global leaders in both pepper cultivation area and yield. As of 2023, the cultivation area in China had expanded to 2.23 million hectares, with a total production of approximately 64 million tons [3,4]. However, conventional pepper production is highly labor-intensive. Specifically, labor costs during the transplanting and harvesting stages account for over 50% of the total production expenses [5]. With the increasing shortage and aging of the agricultural workforce, mechanized production has become an inevitable requirement for the sustainable development of the processing pepper industry [6,7]. Currently, the mechanical harvesting of processing peppers is constrained by technical limitations, such as high rates of fruit damage and fruit drop [8]. These issues primarily arise because most existing processing pepper varieties have been selectively bred with a focus on agronomic traits (e.g., yield, fruit quality, and stress tolerance). Consequently, their plant architecture, biomechanical properties, and population uniformity often fail to match the kinematic mechanisms of agricultural machinery [9]. Therefore, selecting and breeding dedicated pepper varieties suitable for mechanized transplanting and harvesting—thereby aligning crop phenotypes with machine parameters—is crucial for overcoming current technical bottlenecks in the industry [10].

Furthermore, the modern agricultural paradigm is rapidly shifting towards intelligent agricultural systems and field automation. In this broader context, evaluating the machine adaptability of crops is not merely about traditional mechanical matching but also about providing essential prior knowledge for autonomous agricultural robots. Recently, image-based high-throughput plant phenotyping has emerged as a pivotal technological trend, enabling the precise, sensor-driven quantification of complex crop architectures [11]. Concurrently, integrating these phenotypic insights with advanced algorithms (such as deep learning) is revolutionizing the entire crop production chain, driving the transition from automated planting to intelligent harvesting, as successfully demonstrated in other staple crops such as potatoes [12]. Consequently, systematically evaluating the phenotypic and biomechanical adaptability of peppers is a fundamental prerequisite for deploying next-generation intelligent field management systems.

Mechanized operations impose rigorous requirements on the phenotypic and biomechanical traits of peppers. During the transplanting stage, seedlings must exhibit excellent erectness, an optimal hypocotyl length, and robust root cohesion (i.e., a low substrate disintegration rate) to facilitate precise grasping by the mechanical claws of transplanters [13,14,15]. At the harvesting stage, plants require high lodging resistance, an appropriate height of the lowest fruit, clustered fruit-bearing positions, and specific biomechanical properties of the branches. These traits are essential to withstand the stripping or vibrational detachment forces exerted by the harvesting drums [16]. Previous studies have primarily focused on the analysis of single agronomic traits or post-harvest processing quality. However, few investigations have systematically evaluated the integration of morphological features and biomechanical properties across the entire growth cycle, encompassing both seedling transplanting and fruit harvesting [10]. Furthermore, pepper varieties exhibit extensive phenotypic variation, and complex synergistic or antagonistic (trade-off) relationships often exist among various traits. Consequently, traditional screening methods based on single-trait thresholds struggle to objectively and accurately quantify the overall adaptability of these varieties to mechanization.

For the comprehensive evaluation of crop adaptability, previous studies have frequently employed principal component analysis (PCA), the analytic hierarchy process (AHP), or the technique for order preference by similarity to ideal solution (TOPSIS) [17,18]. However, these methods exhibit certain limitations when processing complex, multidimensional mechanization traits. PCA tends to lose specific trait information embedded in minor principal components, and its weight allocation relies solely on mathematical variance, thereby neglecting the practical engineering significance of the indicators [19]. AHP heavily depends on the subjective scoring of experts, which compromises its objectivity [20]. Although TOPSIS maintains objectivity, it cannot effectively address inherent conflicts among evaluation criteria [21]. To eliminate subjectivity and partiality in multidimensional data evaluation, the introduction of multi-criteria decision-making (MCDM) models provides significant rigor. The criteria importance through intercriteria correlation) method (CRITIC) enables objective weighting based on data variability and the conflict among indicators [22]. Meanwhile, the Vlsekriterijumska Optimizacija I Kompromisno Resenje model (VIKOR) effectively manages contradictory evaluation criteria, determining the optimal compromise solution by maximizing group utility and minimizing individual regret [23]. While the CRITIC–VIKOR model has demonstrated high evaluation accuracy in fields such as engineering material optimization, it has not yet been applied to the quantitative evaluation of dedicated crop cultivars for mechanization [24,25].

In this study, an evaluation system comprising 21 key morphological and biomechanical indicators was constructed to assess the suitability of processing peppers for mechanized transplanting and harvesting. Within this framework, a diverse collection of 105 processing pepper varieties (consisting of 56 erect-fruit and 49 pendent-fruit peppers) was quantitatively evaluated across both the seedling and harvesting stages. By employing the coefficient of variation (CV), cluster analysis, PCA, and Pearson correlation analysis, the clustering characteristics of the tested populations and the intrinsic relationships among various traits were revealed. Furthermore, a comprehensive evaluation of the mechanization adaptability of these processing peppers was conducted based on the combined CRITIC–VIKOR decision-making model. The specific objectives of this study were to: (1) establish a quantitative and objective evaluation methodology for the suitability of processing peppers for mechanized transplanting and harvesting; (2) identify the critical limiting traits associated with the mechanization adaptability of processing peppers; and (3) screen and identify processing pepper varieties exhibiting high suitability for mechanization. The findings of this research will provide a theoretical foundation and quantitative model support for the directional breeding of dedicated mechanization-friendly pepper cultivars, as well as the rapid pyramiding of key machine-harvesting traits.

## 2. Materials and Methods

### 2.1. Plant Materials

**Figure 1:**
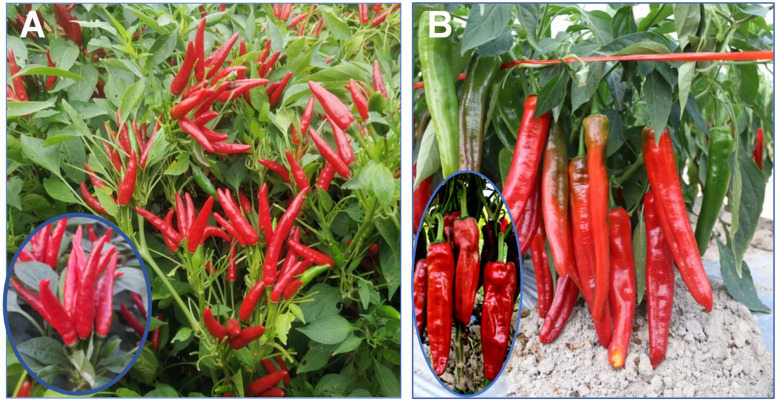
Morphological classification of the 105 processing pepper varieties. (**A**) The erect-fruit pepper group, characterized by upward-pointing fruits. (**B**) The pendent-fruit pepper group, characterized by downward-pointing fruits.

This experiment was conducted from June 2025 to February 2026 at the controlled-environment growth chamber and the experimental field station of the College of Horticulture, Hunan Agricultural University (113°22′ E, 28°29′ N). The tested materials consisted of 105 processing pepper varieties (detailed information and the origins of these varieties are listed in Table S1). The tested materials were divided into two major groups: 56 erect-fruit peppers and 49 pendent-fruit peppers. The erect-fruit pepper group is characterized by upward-pointing fruits (Figure 1A), whereas the pendent-fruit pepper group is characterized by downward-pointing fruits (Figure 1B).

### 2.2. Experimental Design

The experiment was conducted in two sequential stages: seedling cultivation in a growth chamber and plant cultivation in the open field.

*Seedling cultivation stage*: Seedlings were cultivated in 128-cell trays using a commercial substrate provided by Hunan Xianghui Agricultural Technology Development Co., Ltd. (Changsha, China). Seeds were soaked in warm water at 55 °C for 10 min, followed by continuous soaking at room temperature for 6 h. Subsequently, the seeds were germinated in darkness at 28 °C in an incubator. Uniformly germinated seeds were sown at a rate of one seed per cell. Three trays were sown for each variety, with each tray serving as a biological replicate. The trays were placed in a growth chamber with environmental parameters set to 28 °C/18 °C (day/night) and a relative humidity of 60–70%. When over 85% of the seedlings had emerged, a supplemental lighting system was activated with a 12 h/12 h (light/dark) photoperiod and a photosynthetic photon flux density (PPFD) of 200 μmol∙m^−2^∙s^−1^. Once four true leaves had fully expanded, a 0.1% Enshi nutrient solution was applied every 5 days. At 45 days after sowing, robust seedlings with uniform growth were randomly selected from each tray for seedling trait measurements.

*Open-field cultivation stage*: Seedlings at the appropriate stage were transplanted to the experimental field station. The ridges were 17–20 cm high, 60 cm wide at the top, and 80 cm wide at the base, with a distance of 120 cm between adjacent ridges. Plants were transplanted in double rows per ridge with a plant spacing of 40 cm. The experiment was arranged in a randomized complete block design. Each plot covered an area of 24 m^2^ (20 m long × 1.2 m wide), and three replicate plots were established for each variety. Irrigation, fertilization, and pest management were conducted according to standard local cultivation practices. When over 95% of the fruits had reached the red-ripe stage, plant and fruit sampling and measurements were performed.

### 2.3. Measurement of Evaluated Indicators

#### 2.3.1. Seedling Evaluations

**Figure 2:**
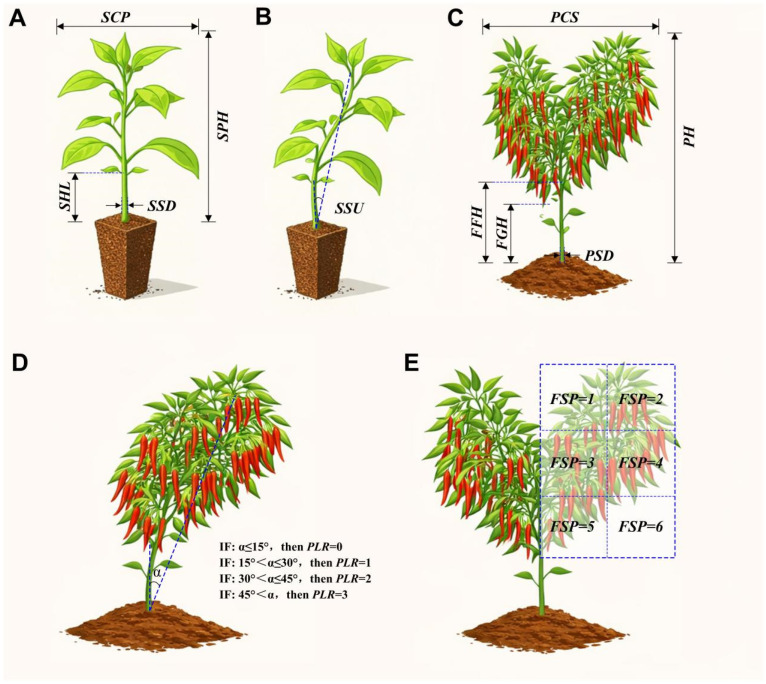
Measurement methods for morphological indicators of processing pepper seedlings and plants. (**A**) Seedling height and canopy spread. (**B**) Stem uprightness of seedlings. (**C**) Plant height and canopy spread. (**D**) Plant lodging resistance. (**E**) Fruit setting position. Note: SPH: Plant height of seedlings; SSD: Stem diameter of seedlings; SHL: Hypocotyl length of seedlings; SCS: Canopy spread of seedlings; SSU: Stem uprightness of seedlings; PH: Plant height; PSD: Plant stem diameter; PCS: Plant canopy spread; FFH: First bifurcation height of plant; PLR: Plant lodging resistance; FSP: Fruit setting position.

Plant height (cm) was measured as the vertical distance from the substrate surface to the apical growing point of the main stem. Stem diameter (mm) was measured as the maximum diameter of the stem at 1 cm below the cotyledonary node using a digital caliper. Canopy spread (cm) was defined as the maximum horizontal width of the plant canopy in its naturally extended state. Hypocotyl length (cm) was measured as the distance from the substrate surface to the cotyledonary node. The measurement methods for these indicators are illustrated in Figure 2A. Erectness (°) was defined as the angle between the vertical line of the main stem and the line connecting the growing point to the stem base, measured using a protractor (Figure 2B).

Stem toughness (MPa): A tensile test was performed on a 5 cm long stem segment using a universal testing machine with an automatic calibration program (Lishi, LE5205, Shanghai, China). The elastic modulus was recorded as the quantitative indicator of stem toughness. Stem hardness (N): A three-point bending test was conducted using the aforementioned universal testing machine. The maximum shear force required to cause yield or fracture at 5 cm from the stem base was recorded. Specifically, the loading speed of the universal testing machine was set to 300 mm/min, and the span length for the three-point bending test was set to 30 mm. To minimize experimental error, nine seedlings of uniform growth vigor were selected per treatment for mechanical property measurements, and the maximum and minimum values in each treatment group were excluded.

This indicator of the substrate disintegration rate of seedlings (SSDR) quantifies the substrate’s resistance to scattering during mechanical grasping and dropping [26]. Five 45-day-old seedlings were randomly selected from each tray for a drop test. The seedlings were allowed to free-fall from a height of 60 cm onto the ground. The scattered substrate (*W*_1_) and the substrate remaining on the roots (*W*_2_) were collected and weighed. The calculation formula is as follows:

$$SSDR(\%)=\frac{W_{1}}{W_{1}+W_{2}}\times 100\%$$

where *W*_1_ is the mass of the scattered substrate (g), *W*_2_ is the mass of the substrate remaining on the roots (g), and (*W*_1_ + *W*_2_) is the total initial mass of the root-ball substrate (g).

#### 2.3.2. Mature Plant Evaluations at Harvest

Plant height, stem diameter, and canopy spread were measured using the same methods as in the seedling stage (Figure 2C). First branch height was measured as the vertical distance from the ground to the first bifurcation of the main stem (Figure 2C). For the above indicators, five plants were randomly selected from each tray/plot for independent measurement, and the average value was taken as one biological replicate. For plant lodging resistance (PLR), ten plants were randomly selected from each plot. The inclination angle (*α*) between the main stem and the ground was measured according to the grading standard shown in Figure 2D. The PLR value was defined as follows: PLR = 0 if α ≤ 15°; PLR = 1 if 15° < α ≤ 30°; PLR = 2 if 30° < α ≤ 45°; and PLR = 3 if α > 45°.

Fruiting branch toughness (N): A fruiting branch from the third bifurcation in the middle of the plant, retaining three internodes, was selected. Both ends of the branch were fixed using a digital push–pull force gauge (DS2-1000N, PUYAN, Taibei, China), and the maximum tensile force required to break the middle internode under uniform stretching was recorded.

#### 2.3.3. Fruit Characteristics

Fruit morphological uniformity (CV, %): Thirty fully colored fruits were randomly selected per plot. ImageJ software 2.14.0 (NIH, Bethesda, MD, USA) was used to extract image features to measure fruit length and diameter [27] and calculate the fruit shape index (length/diameter). The coefficient of variation (CV) of the shape index was used to represent morphological uniformity. Fruit size uniformity (CV, %): The sampling method was the same as described above. The 2D projected area (cm^2^) of each fruit was extracted using ImageJ. The CV of the projected area represented size uniformity. Fruit color parameter (a*): The color parameters of the pericarp were measured using a colorimeter (CHN Spec, DS-200, China). The a* value, representing the red–green axis, was extracted, with a positive value indicating red saturation.

Clearance height of the lowest fruit (cm): Ten plants per plot were randomly selected to measure the vertical distance from the ground to the tip of the lowest fruit. Fruit setting position is defined as the primary distribution interval encompassing the vast majority of effective fruits on a single plant. Briefly, ten plants were randomly selected per plot to capture their two-dimensional side-view canopy profiles. As illustrated by the canopy grid division method in Figure 2E, one-half of the canopy profile was evenly divided into six spatial regions, and the percentage of fruits located within each region was quantified. The specific region harboring the highest proportion of fruits was designated as the fruit setting position and was categorically scored as follows: inner-upper = 1, outer-upper = 2, inner-middle = 3, outer-middle = 4, inner-lower = 5, outer-lower = 6.

Fruit hardness (N): The maximum yield force required for the probe to puncture the pericarp at the equatorial region of the fruit was measured using a fruit penetrometer (Airuipu, GY-4, China). Fruit-pedicel separation force (N): The fruit and pedicel ends were fixed to a digital force gauge to conduct a uniform tensile test. The maximum force at the moment of detachment was recorded. The sample size was nine plants per treatment, and the maximum and minimum measured values were excluded from the final dataset.

### 2.4. Construction of the Comprehensive Evaluation Model

This study integrated the CRITIC weighting method with the VIKOR multi-criteria decision-making model to calculate the group utility value (*S*_i_), individual regret value (*R*_i_), and compromise value (*Q*_i_) for each variety. Ultimately, the varieties were comprehensively ranked regarding their mechanization adaptability based on their *Q*_i_ values.

The CRITIC method determines objective weights based on the contrast intensity and conflict among indicators [28]. The steps are as follows:

**Construction of the original data matrix:** Assuming *n* varieties and *p* evaluation indicators, the original data matrix *X* is formed as follows:

$$X=\left(\begin{array}{ccc} X_{11} & \cdots & X_{1p}\\ \vdots & \ddots & \vdots \\ X_{n1} & \cdots & X_{np} \end{array}\right)$$

where *Xij* represents the value of the *j*-th indicator for the *i*-th variety.

**Data normalization:** To eliminate dimensional effects, Min–Max normalization was applied.

For benefit indicators (the larger, the better):

$$x_{ij}^{\prime }=\frac{{x_{ij}-min(X}_{j})}{{max(X}_{j})-{min(X}_{j})}$$

For cost indicators (the smaller, the better):

$$x_{ij}^{\prime }=\frac{{max(X}_{j}){-x}_{ij}}{{max(X}_{j})-{min(X}_{j})}$$

**Calculation of contrast intensity (*σ*_j_):** Contrast intensity represents the variation of a specific indicator across samples, which is expressed by the standard deviation.

$$\sigma_{j}=\sqrt{\frac{1}{n}\sum_{i=1}^{n}(x_{ij}^{\prime }-{\bar{x}}_{j}{)}^{2}}$$

**Calculation of conflict (*R*_j_):** Conflict is measured based on the correlation coefficient between indicators.

$$R_{j}=\sum_{k=1}^{P}\left(1-r_{jk}\right)$$

where *r*_jk_ is the Pearson correlation coefficient between the j-th and k-th indicators.

**Calculation of information quantity (*C*_j_):** A larger *C*_j_ signifies a greater role of the j-th indicator, warranting a higher weight.

$$C_{j}=\sigma_{j}\times R_{j}$$

**Determination of objective weights (*W*_j_):** The obtained *W*_j_ was subsequently incorporated into the VIKOR model.

$$W_{j}=\frac{C_{j}}{\sum_{j=1}^{p}C_{j}}$$

The VIKOR model is a compromise decision-making method based on ideal solutions [29]. The steps are as follows:

**Determination of ideal solutions:**

For beneficial indicators: $f_{i}^{+}=max(x_{ij})$, $f_{i}^{-}=min(x_{ij})$
For cost indicators: $f_{i}^{+}=min(x_{ij})$, $f_{i}^{-}=max(x_{ij})$

**Calculation of group utility (*S*_i_) and individual regret (*R*_i_):**

$$S_{i}=\sum_{i=1}^{P}\left(W_{i}\frac{f_{i}^{+}-x_{ij}}{f_{i}^{+}-f_{i}^{-}}\right)$$

$$R_{i}=max\left(W_{i}\frac{f_{i}^{+}-x_{ij}}{f_{i}^{+}-f_{i}^{-}}\right)$$

**Calculation of the compromise value (*Q*_i_):**

$$Q_{i}=v\times \frac{S_{i}-S^{+}}{S^{-}-S^{+}}+(1-v)\times \frac{R_{i}-R^{+}}{R^{-}-R^{+}}$$

where *S*^+^ = min(*S*_i_), *S*^−^ = max(*S*_i_), *R*^+^ = min(*R*_i_), and *R*^−^ = max(*R*_i_). The coefficient *v* (0 ≤ *v* ≤ 1) balances group utility and individual regret. In this study, *v* = 0.5 was selected, indicating equal importance for both criteria.

**Comprehensive ranking:** The varieties were ranked based on *Q*_i_. A smaller *Q*_i_ indicates that the variety is closer to the ideal solution, implying superior comprehensive adaptability to mechanized transplanting and harvesting.

### 2.5. Statistical Analysis

Initial data processing was performed using Excel 2019. Multivariate statistical analyses for the 21 indicators were conducted using SPSS 26.0. These analyses included: (1) hierarchical cluster analysis based on Euclidean distance to group the varieties; (2) PCA for dimensionality reduction to identify core factors affecting mechanization adaptability; and (3) Pearson correlation analysis to reveal the synergistic and antagonistic relationships between morphological and biomechanical traits. All statistical graphs were generated using Origin 2022 software.

## 3. Results and Analysis

### 3.1. Construction of the Evaluation Indicator System for the Mechanized Transplanting and Harvesting of Processing Peppers

**Figure 3:**
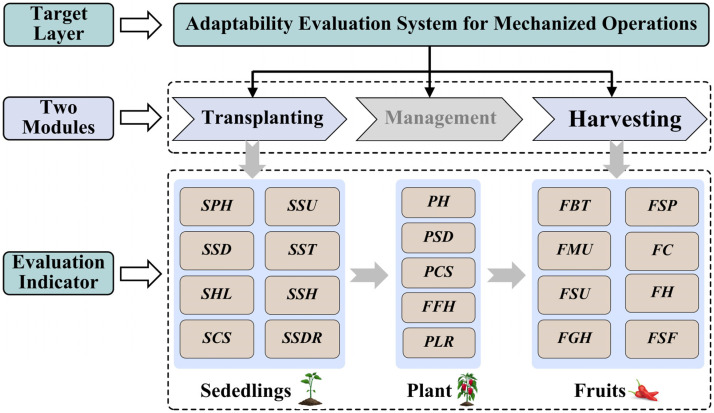
Adaptability evaluation system for mechanized transplanting and harvesting of processing peppers. Note: SPH: Plant height of seedlings; SSD: Stem diameter of seedlings; SHL: Hypocotyl length of seedlings; SCS: Canopy spread of seedlings; SSU: Stem uprightness of seedlings; SST: Stem toughness of seedlings; SSH: Seedlings stem hardness of seedlings; SSDR: Substrate disintegration rate of seedlings; PH: Plant height; PSD: Plant stem diameter; PCS: Plant canopy spread; FFH: First bifurcation height of plant; PLR: Plant lodging resistance; FBT: Fruiting branch toughness; FMU: Fruit morphological uniformity; FSU: Fruit size uniformity; FGH: Lowest fruit height from the ground; FSP: Fruit setting position; FC: Fruit color; FH: Fruit hardness; FSF: Fruit pedicel separation force.

Based on preliminary investigations and open-field tests, this study constructed a comprehensive evaluation indicator system comprising 21 key traits to assess the adaptability of processing peppers to mechanized transplanting and harvesting. The system is divided into two major evaluation modules (Figure 3). The detailed physical significance of each indicator is provided in Table S2, and the specific measurement methods are described in Section 2.3 and Figure 1.

1. *Mechanized transplanting adaptability evaluation module*. This module focuses on the morphological architecture and biomechanical properties of seedlings. It includes eight indicators: plant height (SPH), stem diameter (SSD), hypocotyl length (SHL), canopy spread (SCS), stem uprightness (SSU), stem toughness (SST), stem hardness (SSH), and the substrate disintegration rate of seedlings (SSDR).
2. *Mechanized harvesting adaptability evaluation module*. This module systematically evaluates the mature plant morphology, biomechanical properties, spatial distribution of fruits, and population uniformity. It includes 13 indicators: plant height (PH), stem diameter (PSD), canopy spread (PCS), first bifurcation height (FFH), plant lodging resistance (PLR), fruiting branch toughness (FBT), fruit morphological uniformity (FMU), fruit size uniformity (FSU), lowest fruit height from the ground (FGH), fruit setting position (FSP), fruit color (FC), fruit hardness (FH), and fruit pedicel separation force (FSF).

Of the 21 aforementioned indicators, SPH, SSD, SHL, SSU, SST, SSH, PH, PSD, FFH, FBT, FGH, FSP, FC, and FH were classified as benefit indicators (where higher values are preferable). Conversely, SCS, SSDR, PCS, PLR, FMU, FSU, and FSF were designated as cost indicators (where lower values are preferable). Notably, a higher PLR score corresponds to weaker lodging resistance in the plant; thus, it was logically categorized as a cost indicator.

### 3.2. Variation and Diversity Analysis of Evaluation Indicators

Statistical analysis revealed extensive phenotypic variation across the 21 evaluation indicators among the 105 processing pepper varieties (Table 1 and Table 2, Figure S1). In the erect-fruit pepper group, indicators with large CVs were predominantly concentrated in biomechanical properties and substrate disintegration traits. In descending order, these were PLR (93.60%), SSDR (73.33%), FSF (73.32%), SSU (68.50%), SST (58.30%), SSH (55.53%), and FBT (56.00%), with PLR exhibiting the highest degree of variation. The distribution pattern of high-variation indicators in the pendent-fruit pepper group was highly similar, also reflecting high CVs for SST (58.30%), SSH (64.47%), SSDR (76.06%), PLR (65.72%), FBT (59.00%), and FSF (69.87%). However, in this group, SSDR displayed the maximum variation.

These results indicate that, compared to conventional agronomic traits (e.g., plant height and stem diameter), processing peppers exhibit far more significant variation in mechanical adaptability-related indicators. This phenomenon reflects that traditional pepper breeding objectives have long prioritized conventional traits (such as yield and stress tolerance), thereby exposing a significant imbalance in the compatibility of current cultivars with mechanized operations [10]. Using PLR (the most variable trait) as an example, varieties with poor lodging resistance are highly susceptible to falling over during machine harvesting, which severely reduces harvesting efficiency and increases loss rates.

### 3.3. Cluster Analysis of Processing Pepper Varieties

**Figure 4:**
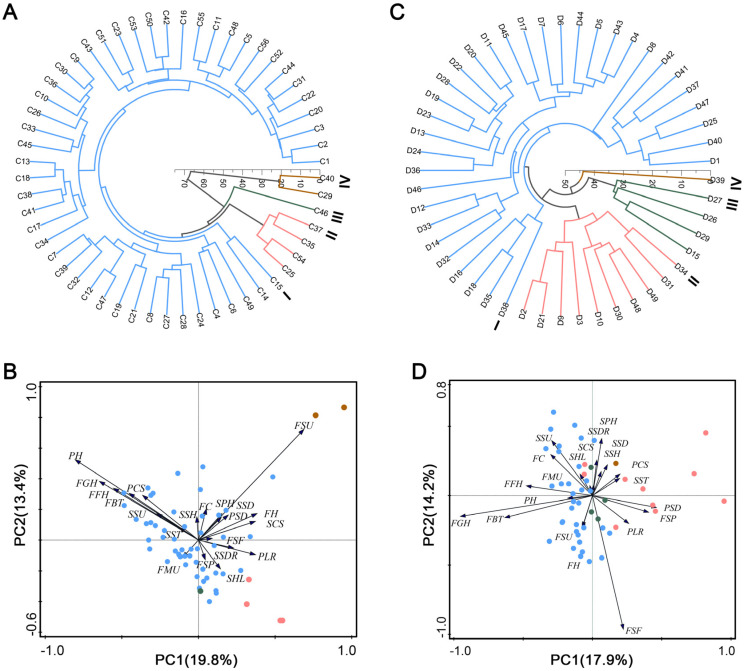
Cluster analysis of the erect-fruit pepper group and the pendent-fruit pepper group. (**A**,**B**) The 56 erect-fruit pepper varieties were divided into four groups. (**C**,**D**) The 49 pendent-fruit pepper varieties were divided into four groups. The colors of the branches (**A**,**C**) and circles (**B**,**D**) represent different clusters, with blue indicating cluster I, red indicating cluster II, green indicating cluster III, and brown indicating cluster IV. Green and red fonts (**B**,**D**) denote traits at the seedling and harvest stages, respectively. SPH: Plant height of seedlings; SSD: Stem diameter of seedlings; SHL: Hypocotyl length of seedlings; SCS: Canopy spread of seedlings; SSU: Stem uprightness of seedlings; SST: Stem toughness of seedlings; SSH: Seedlings stem hardness of seedlings; SSDR: Substrate disintegration rate of seedlings; PH: Plant height; PSD: Plant stem diameter; PCS: Plant canopy spread; FFH: First bifurcation height of plant; PLR: Plant lodging resistance; FBT: Fruiting branch toughness; FMU: Fruit morphologic-al uniformity; FSU: Fruit size uniformity; FGH: Lowest fruit height from the ground; FSP: Fruit setting position; FC: Fruit color; FH: Fruit hardness; FSF: Fruit pedicel separation force.

To reveal the trait distribution characteristics among different groups and provide a basis for screening mechanization-suitable cultivars, a hierarchical cluster analysis based on Euclidean distance was performed on the 56 erect-fruit and 49 pendent-fruit pepper varieties using the 21 indicators. The clustering results are shown in Figure 4.

Within the erect-fruit pepper group (at a Euclidean distance of 42), the 56 tested materials were clustered into four main clades (Figure 4A,B). Cluster I (red branch) is the largest group, containing 49 varieties (e.g., C1–C24 and C26–C28). Its typical characteristics include tall plants, high FFH, high FGH, and tough FBT, but relatively low FH. Cluster II (blue branch) contains four varieties (C25, C35, C37, and C54), characterized by dwarf plants, a compact canopy, and low FFH and fruiting positions. Cluster III (green branch) contains only variety C46, which exhibits a long hypocotyl, excellent lodging resistance, and a distinctive yellow fruit color. Cluster IV (purple branch) includes two varieties (C29 and C40), distinguished by high FH and FSF but poor FSU.

Within the pendent-fruit pepper group (at a Euclidean distance of 37), the 49 materials were also divided into four main clades (Figure 4C,D). Cluster I (red branch), the largest group comprising 34 varieties (e.g., D1 and D4–D8), is characterized by strong FBT, high FGH, and large FSF. Cluster II (blue branch) contains 10 varieties (e.g., D2 and D3), primarily featuring a low FFH and the lowest fruits positioned close to the ground. Cluster III (green branch) includes four varieties (D15, D26, D27, and D29), featuring dwarf plants and a compact canopy but high FH. Cluster IV (purple branch) contains solely variety D39, which displays extreme traits: a long hypocotyl, poor erectness, and fragile seedling stems, coupled with tall plants, a wide canopy, and fragile branches, despite having high fruit hardness.

An ideal processing pepper architecture suitable for mechanical harvesting should integrate multiple traits: a compact plant shape, strong lodging resistance, high ground clearance of the lowest fruits, high branch toughness, and a low pedicel separation force [10,30]. However, the clustering characteristics revealed that none of the eight identified clusters comprehensively met all criteria for the ideal architecture. Each cluster exhibited limitations in specific mechanization-adaptive traits. This fragmented distribution of elite traits highlights the deficiencies in the mechanical adaptability of existing cultivars and provides a direction for future trait-pyramiding breeding.

### 3.4. Principal Component Analysis of Evaluation Indicators

PCA was conducted independently on the 21 indicators for both pepper groups. For the erect-fruit pepper group, eight principal components (PCs) with eigenvalues greater than 1 were extracted, accounting for a cumulative variance contribution rate of 76.01%. For the pendent-fruit pepper group, seven PCs with eigenvalues greater than 1 were extracted, with a cumulative contribution rate of 71.64%. Both models effectively represented the vast majority of the information from the original datasets (Tables S3 and S4), and score plots of PC 1–3 for the two groups of peppers are shown in Figure S2.

**Figure 5:**
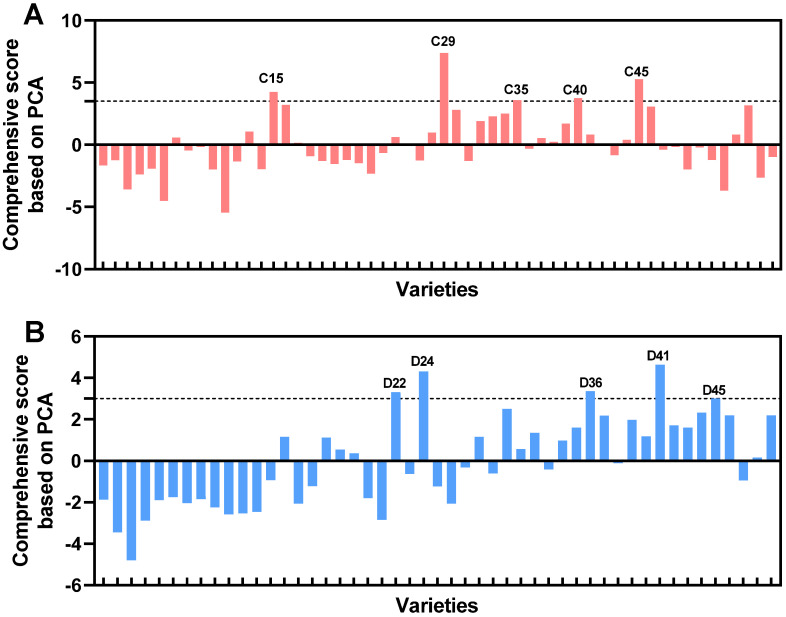
Overall scores for each variety based on the PCA comprehensive evaluation model. The top five varieties are shown for (**A**) the erect-fruit pepper group (C29, C45, C15, C40, and C35) and (**B**) the pendent-fruit pepper group (D41, D24, D36, D22, and D45).

Among the eight PCs of the erect-fruit pepper group, PC1, PC2, PC3, PC4, and PC6 were predominantly associated with the morphological plant architecture traits of seedlings and mature plants. Similarly, in the pendent-fruit pepper group, PC1, PC2, PC3, and PC5 were mainly linked to plant architecture traits. This indicates that architecture-related morphological indicators carry significant weight in the comprehensive evaluation of mechanical performance for both pepper types. Furthermore, indicators related to seedling stem biomechanics, fruit ground clearance, separation force, and substrate disintegration constituted essential secondary PCs. Based on the comprehensive PCA score model, the overall scores for each variety were calculated. The top five varieties in the erect-fruit pepper group were C29, C45, C15, C40, and C35 (Figure 5A). In the pendent-fruit pepper group, the top five were D41, D24, D36, D22, and D45 (Figure 5B).

### 3.5. Correlation Analysis Among Evaluation Indicators

**Figure 6:**
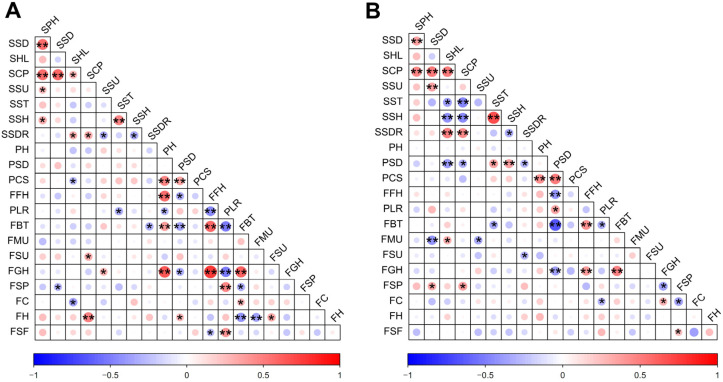
Pearson correlation analysis among the 21 evaluation indicators for (**A**) the erect-fruit pepper group and (**B**) the pendent-fruit pepper group. SPH: Plant height of seedlings; SSD: Stem diameter of seedlings; SHL: Hypocotyl length of seedlings; SCS: Canopy spread of seedlings; SSU: Stem uprightness of seedlings; SST: Stem toughness of seedlings; SSH: Seedlings stem hardness of seedlings; SSDR: Substrate disintegration rate of seedlings; PH: Plant height; PSD: Plant stem diameter; PCS: Plant canopy spread; FFH: First bifurcation height of plant; PLR: Plant lodging resistance; FBT: Fruiting branch toughness; FMU: Fruit morphologic-al uniformity; FSU: Fruit size uniformity; FGH: Lowest fruit height from the ground; FSP: Fruit setting position; FC: Fruit color; FH: Fruit hardness; FSF: Fruit pedicel separation force. “*” represents a significant difference at *p* < 0.05, and “**” at *p* < 0.01.

To explore the potential intrinsic associations among phenotypic and biomechanical traits, and to provide a theoretical basis for directional breeding and trait pyramiding, a Pearson correlation analysis was performed on the 21 indicators for both groups. The results demonstrated that the two groups shared highly synergistic commonalities while also exhibiting significant population heterogeneity in trait evolution (Figure 6).

*Common traits across both groups*: At the seedling stage, highly significant positive correlations were observed among SPH, SSD, and SCS. However, SSD showed no direct significant correlation with SST or SSH. Notably, SSDR was significantly positively correlated with SHL and SCS. This suggests that seedlings with overly vigorous above-ground vegetative growth possess relatively weaker below-ground root cohesion, making their root balls more prone to scattering upon dropping during mechanical transplanting. At the mature stage, PCS was significantly positively correlated with PH and PSD. Additionally, FGH showed a significant positive correlation with FFH and FBT. Interestingly, FBT was significantly negatively correlated with PSD and the PLR score (note that a lower PLR score indicates stronger lodging resistance), which implies that plants with tougher fruiting branches generally possess higher fruit ground clearance and stronger lodging resistance—a trait combination highly favorable for mechanical harvesting.

*Differentiating traits between the groups*: In the pendent-fruit pepper group, seedling SSH was significantly negatively correlated with SHL and SCS, indicating that more vigorous pendent-fruit pepper seedlings paradoxically develop more fragile stems (reduced SSH). This trend was not observed in the erect-fruit pepper group. Furthermore, in the erect-fruit pepper group, FGH was significantly positively correlated with PH; however, due to the large variation and interference of fruit length in the pendent-fruit pepper group, no such correlation was found. Additionally, in the erect-fruit pepper group, FSP was significantly positively correlated with the PLR score (fruits located more toward the outer canopy resulted in poorer lodging resistance), whereas the pendent-fruit pepper group showed no clear association for this trait.

### 3.6. Comprehensive Evaluation of Suitability for Mechanized Transplanting and Harvesting

To eliminate the partiality inherent in single-indicator evaluations, this study integrated the CRITIC weighting method with the VIKOR multi-criteria decision-making model to conduct a comprehensive quantitative evaluation of mechanization suitability for both groups.

**Figure 7:**
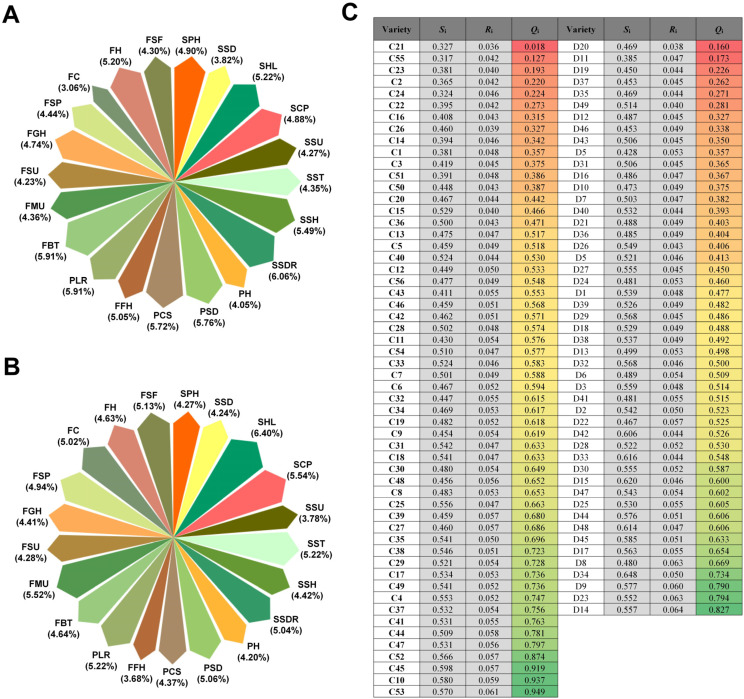
Comprehensive evaluation of suitability for mechanized transplanting and harvesting using the CRITIC–VIKOR model. (**A**,**B**) Objective weighting of the 21 indicators using the CRITIC method for (**A**) the erect-fruit pepper group and (**B**) the pendent-fruit pepper group. (**C**) VIKOR model-based comprehensive evaluation of the 56 erect-fruit pepper and 49 pendent-fruit pepper varieties. Colors denote the *Q*_i_ values, which reflect the mechanized transplanting and harvesting performance of the tested cultivars. Red indicates lower *Q*_i_ values (better performance), while green indicates higher *Q*_i_ values (poorer performance). SPH: Plant height of seedlings; SSD: Stem diameter of seedlings; SHL: Hypocotyl length of seedlings; SCS: Canopy spread of seedlings; SSU: Stem uprightness of seedlings; SST: Stem toughness of seedlings; SSH: Seedlings stem hardness of seedlings; SSDR: Substrate disintegration rate of seedlings; PH: Plant height; PSD: Plant stem diameter; PCS: Plant canopy spread; FFH: First bifurcation height of plant; PLR: Plant lodging resistance; FBT: Fruiting branch toughness; FMU: Fruit morphologic-al uniformity; FSU: Fruit size uniformity; FGH: Lowest fruit height from the ground; FSP: Fruit setting position; FC: Fruit color; FH: Fruit hardness; FSF: Fruit pedicel separation force. *S*_i_: Calculation of group utility. *R*_i_: individual regret. *Q*_i_: compromise value. A smaller *Q*_i_ indicates that the variety is closer to the ideal solution.

Objective weighting of the 21 indicators was performed using the CRITIC method (Figure 7A,B). For the erect-fruit pepper group, the top four core indicators with the highest weight proportions were SSDR (6.06%), FBT (5.91%), PSD (5.76%), and PCS (5.72%). This indicates that transplanting root-ball integrity, harvest-stage branch fracture resistance, stem support strength, and plant canopy compactness collectively form the core trait cluster determining the mechanical adaptability of erect-fruit peppers. Moreover, the weights for SSH, SHL, FH, and PLR all exceeded 5.000%. For the pendent-fruit pepper group, the top three indicators were SHL (6.396%), SCS (5.541%), and FMU (5.521%), revealing that seedling compactness and fruit morphological consistency are the key limiting factors for their mechanization. Indicators such as SST, PLR, and FSF also exceeded 5.000% in weight, serving as crucial auxiliary evaluation parameters.

Based on these objective weights, the comprehensive compromise values (*Q*_i_) for all tested varieties were calculated using the VIKOR model, and the varieties were subsequently ranked (Figure 7C). The results indicated that varieties C21, C55, and C23 in the erect-fruit pepper group, and D20, D11, and D19 in the pendent-fruit pepper group, obtained the smallest *Q*_i_ values. These varieties exhibited superior comprehensive adaptability to mechanized transplanting and harvesting and can be recommended as primary candidate cultivars for mechanized production. Simultaneously, varieties C2, C24, and C22 (erect-fruit peppers), as well as D37, D35, and D49 (pendent-fruit peppers), demonstrated excellent overall performance across key mechanization traits, making them highly promising secondary candidates.

## 4. Discussion

### 4.1. Variation and Trade-Off Effects of Key Traits in Mechanization Adaptability

Mechanized transplanting and harvesting require a comprehensive evaluation of population uniformity and biomechanical properties, rather than merely screening the morphological phenotypes of individual plants [31,32]. This study found that in both the erect-fruit and pendent-fruit pepper groups, the CVs for biomechanical properties (e.g., PLR, SSH, and FSF) and substrate disintegration rate (SSDR) were considerably higher than those for conventional morphological indicators. This result indicates immense potential for improving the biomechanical properties of current processing pepper varieties. Furthermore, it confirms that traditional breeding has long prioritized yield and stress tolerance while relatively neglecting plant biomechanics, resulting in high vulnerability and instability when existing cultivars are subjected to external mechanical forces, such as dropping, combing, and striking [33,34,35]. Previous studies have established a strong statistical correlation between poor plant lodging resistance and significantly increased fruit damage rates, as well as severe yield losses during mechanized operations [36,37]. Therefore, the high variation in plant lodging resistance (PLR) observed in our study directly underscores its critical role as a limiting factor in actual mechanized harvesting performance, emphasizing the necessity of targeted biomechanical improvement.

Cluster and correlation analyses further revealed complex “trade-off” effects between agronomic traits and biomechanical characteristics. For instance, the FGH was significantly positively correlated with the FFH and FBT, which is highly beneficial for the chassis clearance and combing efficiency of mechanical harvesters. However, the elevation of FFH inevitably raises the plant’s overall center of gravity, thereby exhibiting a significant negative correlation with PLR. Similarly, at the seedling stage, seedlings with a larger SCS and above-ground biomass often exhibited a higher SSDR. This reflects resource competition between above-ground growth and below-ground root development; excessive above-ground growth leads to relatively insufficient root penetration and cohesion in the substrate. The clustering results also corroborated this antagonistic mechanism. Among the 105 tested varieties, no single cluster could comprehensively meet all the ideal criteria for mechanized operations (e.g., Cluster I of the erect-fruit peppers had tough FBT but low FH; Cluster IV had high FH but poor FSU). These mutual constraints among traits demonstrate that achieving a high level of mechanization adaptability through single-trait improvement is unrealistic. Future directional breeding must be based on the co-evolution of multiple traits to achieve rapid trait pyramiding [35].

### 4.2. Differences in Mechanization Adaptability Between the Two Pepper Groups

There are fundamental differences in the spatial distribution of fruits between erect-fruit and pendent-fruit peppers, which justify their independent classification and evaluation in this study. Both multivariate statistical analyses and CRITIC objective weighting confirmed that the core adaptive characteristics of the two groups differ significantly in response to mechanized transplanting and harvesting [38].

In the erect-fruit pepper group, fruits are predominantly clustered and grow upright, and mechanical harvesting primarily utilizes drum striking or combing [10]. Consequently, the CRITIC weighting model objectively assigned extremely high weights to FBT (5.91%) and PSD (5.76%) at the harvest stage. In contrast, the fruits of the pendent-fruit pepper group hang vertically downward, with a relatively dispersed gravity distribution and significant variation in fruit length. Correlation analysis revealed that FGH in this group is primarily determined by the downward hanging length of the fruit, showing a low correlation with plant height itself. This morphological feature shifts the key limiting factor for mechanized harvesting adaptability toward fruit uniformity. The CRITIC analysis corroborated this, showing that in the pendent-fruit pepper group, FMU (5.52%) replaced FBT as the core factor ensuring mechanical picking quality and reducing omission and damage rates. These significant differences provide a quantitative basis for the deep integration of agricultural machinery and agronomy. Directional breeding and machinery design for erect-fruit peppers should focus on high FBT and PLR, whereas for pendent-fruit peppers, the emphasis should be on selecting varieties with high FMU and uniform hanging lengths.

### 4.3. Superiority and Application Prospects of the CRITIC–VIKOR Comprehensive Evaluation Model

The combined CRITIC–VIKOR model introduced in this study effectively resolves the subjective challenges of weight allocation and multidimensional indicator conflicts in the mechanization evaluation of processing peppers. The CRITIC method relies entirely on the variability and conflict of the data itself for objective weighting, thereby avoiding the interference of human empirical judgment [38]. The core advantage of the VIKOR model lies in its ranking mechanism based on “compromise programming”, which maximizes the group utility value (*S*_i_) while strictly constraining the individual regret value (*R*_i_) [39,40]. To quantitatively demonstrate the superiority of the CRITIC–VIKOR model over traditional PCA, comparative performance metrics were analyzed. The highest value of core biomechanical traits (e.g., PLR) among the VIKOR-selected Top 10 of erect-fruit peppers was 0.27, and lower than that of the PCA-selected top 10 (2.23), indicating that VIKOR possesses a stronger discriminatory power for screening elite cultivars.

Taking variety C29 in this study as an example: if evaluated solely based on traditional PCA comprehensive scores, this variety might mask its severe deficiencies in specific biomechanical traits due to outstanding performance in certain morphological indicators, even ranking first in the comprehensive score (Figure 5). However, in the CRITIC–VIKOR evaluation system, C29’s final ranking dropped significantly. The mathematical mechanism driving this evaluation contrast is that the CRITIC–VIKOR model first amplifies the influence of core mechanization indicators (e.g., PLR) through objective weighting. Subsequently, even if C29 performs adequately in most morphological indicators, its severe defects in individual key biomechanical indicators generate a massive “individual regret value (*R*_i_)”. This extreme disadvantage in a single indicator translates into a strong penalty in the VIKOR model, ultimately leading to a significant increase in its comprehensive compromise value (*Q*_i_) and a drastic drop in its overall ranking.

This strict constraint mechanism of the VIKOR model is highly consistent with the logic of “Liebig’s Law of the Minimum” in actual agricultural mechanized production [41,42]. In a real machine-harvesting scenario, regardless of how uniform the fruit size is or how high the fruit yield per plant is, if the plant experiences extensive lodging or the main branches break easily, the resulting fruit damage and yield loss will drastically increase. Consequently, the mechanical harvesting rate will sharply decline or even drop to zero, rendering advantages in other morphological indicators practically meaningless for engineering purposes [43,44]. Therefore, the superior erect-fruit pepper varieties (C21, C55, and C23) and pendent-fruit pepper varieties (D20, D11, and D19) ultimately screened by this model not only perform excellently in overall transplanting and harvesting traits but, more importantly, effectively avoid single “fatal” flaws. Furthermore, the elite varieties identified through model validation require further field trials for transplanting and harvesting. Their performance will be evaluated based on transplant survival rates and harvest loss rates. These corresponding validation experiments will be conducted in subsequent studies. This study proposes a strategy of “model-guiding combined with field validation”. The model is first utilized to conduct assessment analysis on an extensive collection of varieties. Then, empirical field trials are focused specifically on the most promising mechanization-suitable cultivars. This approach not only ensures the scientific reliability of the evaluation results but also substantially reduces the time and economic costs associated with large-scale field testing.

### 4.4. Practical Implications and Future Perspectives for Intelligent Agricultural Systems

The systematic evaluation framework constructed in this study provides profound practical implications for guiding cultivar selection, agronomic–machine matching, and future intelligent field management. From a breeding perspective, the objective weights determined by the CRITIC model provide clear quantitative targets for trait pyramiding. From a machinery design perspective, the biomechanical thresholds of the top-ranked varieties (such as FBT and FSF) supply essential engineering parameters for setting the kinematic mechanisms of harvesting drums and transplanting claws, thereby bridging the gap between crop biology and machinery engineering.

Furthermore, beyond conventional mechanized production, the modern agricultural paradigm is rapidly advancing toward autonomous operations and field robotics. In this context, visual navigation is a promising future direction for agricultural mechanization and field robotics because robust perception and navigation are essential for linking plant architecture traits with machine operation strategies [45]. Although the primary focus of this study is matching physical crop traits to mechanical requirements, it is highly relevant to the design of future intelligent agricultural robots. A highly standardized plant population (characterized by high lodging resistance, clustered fruit-bearing positions, and consistent lowest-fruit clearance height) significantly reduces the background noise and algorithmic complexity required for machine vision processing in complex open-field environments. Consequently, the suitability evaluation of plant architecture and biomechanical traits conducted in this study not only solves current mechanization bottlenecks but also provides crucial prior knowledge for visual navigation, target perception, and end-effector design in autonomous agricultural systems. By ensuring that pepper cultivars are inherently “robot-friendly,” this evaluation system lays a robust biological foundation for the development of next-generation intelligent field management.

## 5. Conclusions

This study systematically constructed an adaptability evaluation system for the mechanized operations of processing peppers, encompassing both seedling and harvesting stages and integrating agronomic morphology with biomechanical properties. Variation analysis, hierarchical clustering, PCA, and Pearson correlation analysis revealed extremely high variation in the biomechanical properties of the tested pepper varieties. Furthermore, significant “trade-off” effects were identified among key mechanization traits; for example, an increase in FFH inevitably led to a decrease in PLR and an enlarged SCS exacerbated SSDR. These antagonistic constraints demonstrate the necessity of introducing multi-criteria decision-making models in the breeding of cultivars for mechanical harvesting. CRITIC objective weighting analysis indicated significant divergence in the mechanization-limiting factors between the two groups: the adaptability of erect-fruit peppers is primarily limited by fruiting branch toughness, whereas pendent-fruit peppers are more constrained by fruit morphological uniformity. The introduced CRITIC–VIKOR multi-criteria evaluation model effectively overcame the drawback of the traditional single PCA score, which can mask individual fatal flaws, thereby preventing the negative impact of single critical biomechanical defects on mechanical operations. Utilizing this model, six elite varieties were successfully screened: C21, C55, and C23 (erect-fruit pepper group) and D20, D11, and D19 (pendent-fruit pepper group). These varieties exhibited excellent comprehensive synergy in morphology and biomechanics during both transplanting and harvesting stages, without obvious shortcomings in mechanization adaptability, making them prime candidates for future large-scale mechanized production.

## Figures and Tables

**Table 1:** Variation and diversity analysis of evaluation indicators for 56 erect-fruit pepper varieties.

Stage	Evaluation Indicators	Indicator Type	Maximum Values	Minimum Values	Mean Values	Standard Deviation	Coefficient of Variation%
Transplanting	SPH (cm)	Benefit	17.37	5.11	11.85	2.80	23.61
	SSD (mm)	Benefit	3.22	1.26	2.24	0.34	15.03
	SHL (cm)	Benefit	6.54	1.07	2.99	1.22	40.76
	SCS (cm)	Cost	18.99	8.67	12.10	2.21	18.30
	SSU (°)	Benefit	36.14	1.48	10.24	7.02	68.50
	SST (MPa)	Benefit	2.82	0.29	0.98	0.54	54.50
	SSH (N)	Benefit	0.86	0.04	0.39	0.22	55.53
	SSDR (%)	Cost	0.50	0.01	0.20	0.15	73.33
Harvesting	PH (cm)	Benefit	92.83	37.91	66.69	11.13	16.68
	PSD (cm)	Benefit	13.90	7.69	11.08	1.54	13.87
	PCS (cm)	Cost	83.30	41.11	64.43	10.49	16.28
	FFH (cm)	Benefit	37.78	11.93	24.11	5.63	23.36
	PLR	Cost	2.23	0.00	0.61	0.57	93.60
	FBT (N)	Benefit	24.56	1.35	12.20	6.83	56.00
	FMU (%)	Cost	40.19	6.47	18.89	6.67	35.31
	FSU (%)	Cost	89.42	18.36	30.18	13.57	44.94
	FGH (cm)	Benefit	47.99	15.77	30.66	8.04	26.24
	FSP	Benefit	4.00	1.33	2.32	0.48	20.77
	FC	Benefit	31.88	−3.18	26.80	4.93	18.40
	FH (N)	Benefit	38.05	13.86	23.86	5.38	22.53
	FSF (N)	Cost	18.22	0.79	4.88	3.58	73.32

Note: SPH: Seedling plant height, SSD: Seedling stem diameter, SHL: Seedling hypocotyl length, SCS: Seedling canopy spread, SSU: Seedling stem uprightness, SST: Seedling stem toughness, SSH: Seedling stem hardness, SSDR: Substrate disintegration rate of seedlings, PH: Plant height, PSD: Plant stem diameter, PCS: Plant canopy spread, FFH: First bifurcation height, PLR: Plant lodging resistance, FBT: Fruiting branch toughness, FMU: Fruit morphological uniformity, FSU: Fruit size uniformity, FGH: Lowest fruit height from the ground, FSP: Fruit setting position, FC: Fruit color, FH: Fruit hardness, FSF: Fruit pedicel separation force.

**Table 2:** Variation and diversity analysis of evaluation indicators for 49 pendent-fruit pepper varieties.

Stage	Evaluation Indicators	Indicator Type	Maximum Values	Minimum Values	Mean Values	Standard Deviation	Coefficient of Variation%
Transplanting	SPH (cm)	Benefit	17.54	7.76	12.12	2.18	17.95
	SSD (mm)	Benefit	3.19	1.61	2.23	0.32	14.41
	SHL (cm)	Benefit	6.08	1.18	3.46	1.40	40.45
	SCS (cm)	Cost	16.61	8.69	12.26	2.07	16.88
	SSU (°)	Benefit	23.74	2.01	8.66	4.22	48.79
	SST (MPa)	Benefit	2.52	0.23	1.02	0.59	58.30
	SSH (N)	Benefit	1.29	0.08	0.43	0.28	64.47
	SSDR (%)	Cost	0.59	0.00	0.19	0.14	76.06
Harvesting	PH (cm)	Benefit	90.16	44.42	62.64	9.71	15.50
	PSD (cm)	Benefit	16.87	7.78	11.65	2.07	17.72
	PCS (cm)	Cost	92.08	47.46	68.93	9.07	13.16
	FFH (cm)	Benefit	37.93	12.84	22.27	4.76	21.38
	PLR	Cost	2.40	0.00	0.97	0.64	65.72
	FBT (N)	Benefit	27.93	1.74	10.23	6.03	59.00
	FMU (%)	Cost	41.19	10.12	22.59	8.66	38.36
	FSU (%)	Cost	63.09	11.99	30.50	11.01	36.10
	FGH (cm)	Benefit	44.23	0.00	23.70	9.84	41.50
	FSP	Benefit	4.44	1.78	2.85	0.60	21.06
	FC	Benefit	31.62	16.85	25.36	3.82	15.07
	FH (N)	Benefit	34.48	13.54	20.95	4.67	22.29
	FSF (N)	Cost	23.90	0.54	8.52	5.95	69.87

## Data Availability

The original contributions presented in this study are included in the article. Further inquiries can be directed to the corresponding author.

## Supplementary Materials

The following supporting information can be downloaded at: https://www.mdpi.com/article/10.3390/plants15101441/s1, Table S1: Names, codes and sources of 105 tested processing pepper varieties; Table S2: Evaluation indicators for suitability of processing peppers to mechanized transplanting and harvesting; Table S3: Principal component analysis of evaluation indicators for suitability of chili peppers to mechanized transplanting and harvesting; Table S4: Principal component analysis of evaluation indicators for suitability of plate peppers to mechanized transplanting and harvesting; Figure S1: Variation and diversity analysis of evaluation indicators for 105 processing peppers varieties; Figure S2: Score plots of principal components 1–3 based on principal component analysis.
