# Supplementary material for: Construction of an Evaluation System and Comprehensive Assessment of the Suitability of Different Processing Peppers for Mechanized Transplanting and Harvesting
Source: Plants (Basel). 2026 May 8;15(10):1441. doi: 10.3390/plants15101441 (PMC13210977; doi:10.3390/plants15101441)
Supplement: Supplementary file 1 [file plants-15-01441-s001.zip › plants-4272661-supplementary.pdf]

Table S1 Names, codes and sources of 105 tested processing pepper varieties

Type	Number	Variety Name	Source	Type	Number	Variety Name	Source
Chili peppers	C1	Bola Tianjiao No. 1	Hunan Xingshu Seed Industry Co., Ltd.	Plate peppers	D1	Bola Zhouxian No. 1	Hunan Xingshu Seed Industry Co., Ltd.
	C2	Bola Tianjiao No. 2	Hunan Xingshu Seed Industry Co., Ltd.		D2	Bola Zhouxian No. 3	Hunan Xingshu Seed Industry Co., Ltd.
	C3	Bola Tianjiao No. 4	Hunan Xingshu Seed Industry Co., Ltd.		D3	Bola Zhouxian No. 6	Hunan Xingshu Seed Industry Co., Ltd.
	C4	Bola Tianjiao 60	Hunan Xingshu Seed Industry Co., Ltd.		D4	Bola Hongniu	Hunan Xingshu Seed Industry Co., Ltd.
	C5	Bola Caomeijiao No. 1	Hunan Xingshu Seed Industry Co., Ltd.		D5	Bola Hongshuai	Hunan Xingshu Seed Industry Co., Ltd.
	C6	Bola Caomeijiao No. 2	Hunan Xingshu Seed Industry Co., Ltd.		D6	Xingshu Huanggongjiao No. 2	Hunan Xingshu Seed Industry Co., Ltd.
	C7	Bola Caomeijiao No. 11	Hunan Xingshu Seed Industry Co., Ltd.		D7	Bola No. 15	Hunan Xingshu Seed Industry Co., Ltd.
	C8	Bola Tianxing 712	Hunan Xingshu Seed Industry Co., Ltd.		D8	Xiangganla No. 1	Hunan Xingshu Seed Industry Co., Ltd.
	C9	Bola Tianyu	Hunan Xingshu Seed Industry Co., Ltd.		D9	Manjianghong	Hunan Xingshu Seed Industry Co., Ltd.
	C10	Bola Tianyu No. 2	Hunan Xingshu Seed Industry Co., Ltd.		D10	Changla No. 7	Changsha Chunrun Seed Industry Co., Ltd.
	C11	Bola Tianyu No. 4	Hunan Xingshu Seed Industry Co., Ltd.		D11	Changla No. 17	Changsha Chunrun Seed Industry Co., Ltd.

C12	Bola Tianyu No. 6	Hunan Xingshu Seed Industry Co., Ltd.	D12	Changla No. 27	Changsha Chunrun Seed Industry Co., Ltd.
C13	Bola Tianyu No. 8	Hunan Xingshu Seed Industry Co., Ltd.	D13	Xiangshuala No. 3	Hunan Xiangyan Seed Industry Co., Ltd.
C14	Bola Tianyu No. 11	Hunan Xingshu Seed Industry Co., Ltd.	D14	Xiangshuala No. 7	Hunan Xiangyan Seed Industry Co., Ltd.
C15	Xiangla 107	Hunan Xiangyan Seed Industry Co., Ltd.	D15	Xiangyanhong No. 2	Hunan Xiangyan Seed Industry Co., Ltd.
C16	Xiangyanhongzhua	Hunan Xiangyan Seed Industry Co., Ltd.	D16	Xiangshuala No. 8	Hunan Xiangyan Seed Industry Co., Ltd.
C17	Xiangla 762	Hunan Xiangyan Seed Industry Co., Ltd.	D17	Xiangyanhong No. 1	Hunan Xiangyan Seed Industry Co., Ltd.
C18	Xiangla 712	Hunan Xiangyan Seed Industry Co., Ltd.	D18	Shangla Ganxian No. 2	Anhui Shanggao Agricultural Technology Co., Ltd.
C19	Xiangla 1417	Hunan Xiangyan Seed Industry Co., Ltd.	D19	Shangla Xichangxian	Anhui Shanggao Agricultural Technology Co., Ltd.
C20	Xiangla 702	Hunan Xiangyan Seed Industry Co., Ltd.	D20	Hongguan 603	Hunan Xiangmeizi Agricultural Technology Co., Ltd.
C21	Xiangla 52	Hunan Xiangyan Seed Industry Co., Ltd.	D21	Xiangla Jichang	Hunan Xiangmeizi Agricultural Technology Co., Ltd.
C22	Xiangla 54	Hunan Xiangyan Seed Industry Co., Ltd.	D22	Haimai 3000S Line Pepper	Sichuan Haimai Seed Industry Co., Ltd.

C23	Hongzhua No. 2	Hunan Xiangyan Seed Industry Co., Ltd.	D23	Bentian Line Pepper	Sichuan Haimai Seed Industry Co., Ltd.
C24	Xiangla 57	Hunan Xiangyan Seed Industry Co., Ltd.	D24	Hongpaojian New Pointed Pepper	Sichuan Haimai Seed Industry Co., Ltd.
C25	Hongzhua No. 6	Hunan Xiangyan Seed Industry Co., Ltd.	D25	Haimai PC20 Line Pepper	Sichuan Haimai Seed Industry Co., Ltd.
C26	Hongzhua No. 7	Hunan Xiangyan Seed Industry Co., Ltd.	D26	Haimai Changjiao 20 Line Pepper	Sichuan Haimai Seed Industry Co., Ltd.
C27	Guifei F1	Hangzhou Sanjiang Seed Industry Co., Ltd.	D27	Lvyanghe L11 Line Pepper	Sichuan Haimai Seed Industry Co., Ltd.
C28	Shanjiao Wentian No. 2	Anhui Shanggao Agricultural Technology Co., Ltd.	D28	Haimai Hong 200	Sichuan Haimai Seed Industry Co., Ltd.
C29	Yuehong 462	Jiangxi Yuefeng Seed Industry Co., Ltd.	D29	Yanjiao No. 11	Chongqing Keguang Seedling Co., Ltd.
C30	Yuefeng Yushuo	Jiangxi Yuefeng Seed Industry Co., Ltd.	D30	Yanjiao No. 15	Chongqing Keguang Seedling Co., Ltd.
C31	Tianhuo No. 2 Capsicum annuum	Sichuan Haimai Seed Industry Co., Ltd.	D31	Yanjiao No. 16	Chongqing Keguang Seedling Co., Ltd.
C32	Haimai Hong 2588	Sichuan Haimai Seed Industry Co., Ltd.	D32	Yujiao No. 13	Chongqing Keguang Seedling Co., Ltd.
C33	Shengjiao No. 3	Chongqing Keguang Seedling Co., Ltd.	D33	Yujiao No. 15	Chongqing Keguang Seedling Co., Ltd.
C34	Yanjiao 425	Chongqing Keguang Seedling Co., Ltd.	D34	Yujiao No. 5	Chongqing Keguang Seedling Co., Ltd.
C35	Yanjiao 435	Chongqing Keguang Seedling Co., Ltd.	D35	Yunganjiao No. 8	Yunnan Academy of Agricultural Sciences

C36	Yanjiao 465	Chongqing Keguang Seedling Co., Ltd.	D36	Yunganjiao No. 9	Yunnan Academy of Agricultural Sciences
C37	Yanjiao 475	Chongqing Keguang Seedling Co., Ltd.	D37	Yunganjiao No. 10	Yunnan Academy of Agricultural Sciences
C38	Yanjiao 485	Chongqing Keguang Seedling Co., Ltd.	D38	Yunganjiao No. 11	Yunnan Academy of Agricultural Sciences
C39	Yanjiao 538	Chongqing Keguang Seedling Co., Ltd.	D39	Yunganjiao No. 14	Yunnan Academy of Agricultural Sciences
C40	Yanjiao 548	Chongqing Keguang Seedling Co., Ltd.	D40	Ganxian No. 1	Huaibei Jiubotian Seed Industry Co., Ltd.
C41	Yanjiao 800	Chongqing Keguang Seedling Co., Ltd.	D41	Dajinhong	Huaibei Jiubotian Seed Industry Co., Ltd.
C42	Yunganjiao No. 7	Yunnan Academy of Agricultural Sciences	D42	Jiuyan No. 22	Huaibei Jiubotian Seed Industry Co., Ltd.
C43	Jiuyan No. 5	Huaibei Jiubotian Seed Industry Co., Ltd.	D43	Hongli No. 1	Huaibei Jiubotian Seed Industry Co., Ltd.
C44	Lamei No. 4	Huaibei Jiubotian Seed Industry Co., Ltd.	D44	Jiuyan No. 27	Huaibei Jiubotian Seed Industry Co., Ltd.
C45	Xinmantian 105	Huaibei Jiubotian Seed Industry Co., Ltd.	D45	Fengyan No. 21	Huaibei Jiubotian Seed Industry Co., Ltd.
C46	Yulinglong	Huaibei Jiubotian Seed Industry Co., Ltd.	D46	Yiduhong Pepper	Qingxian Chunfeng Seed Industry Co., Ltd.
C47	Hongla No. 1	Huaibei Jiubotian Seed Industry Co., Ltd.	D47	Layan No. 517	Guizhou Academy of Agricultural Sciences

C48	Xinmantian No. 1	Huaibei Jiubotian Seed Industry Co., Ltd.	D48	Honglong No. 23	Xinjiang Tianjiao Hong'an Agricultural Technology Co., Ltd.
C49	Japanese Capsicum annuum var.	Harbin Jinlong Agriculture Co., Ltd.	D49	Hongyuan 15	Qingdao Sanhe Agricultural Technology Co., Ltd.
C50	Chiyan No. 6	Shouguang Xinxinran Horticulture Co., Ltd.			
C51	Chiyan No. 2	Shouguang Xinxinran Horticulture Co., Ltd.			
C52	Yuanzhong Caotianjiao	Qingxian Qianrui Agricultural Technology Co., Ltd.			
C53	Neihuang New Generation	Dingzhou Nongle Seed Industry Co., Ltd.			
C54	Layan No. 112	Guizhou Academy of Agricultural Sciences			
C55	Huanggu 882	Liaoning Yijuyang Seedling Co., Ltd.			
C56	Hongsheng No. 1	Liaoning Yijiuxiang Seed & Seedling Co., Ltd.			

Table S2 Evaluation indicators for suitability of processing peppers to mechanized transplanting and harvesting

Number	Stage	Indicator Name	Abbre -viation	Indicator Description
1	Transplan-ting stage	Plant height of seedlings	SPH	SPH is a fundamental parameter for the path planning and positioning of the robotic manipulator during the grasping process. Uniformity in plant height standardizes grasping depth and force, thereby improving operational efficiency. Excessively tall plants are prone to breakage or lodging during transportation and grasping. Conversely, if plants are too short, accurate localization by the manipulator becomes difficult. Inconsistent plant heights directly increase the rates of missed transplantations and seedling damage.
2		Stem diameter of seedlings	SSD	SSD directly reflects the mechanical strength of the seedling. A thicker stem can withstand the pressure applied during mechanical clamping, reducing the risk of injury or breakage and ensuring seedling survival and rapid recovery post-transplantation.
3		Hypocotyl length of seedlings	SHL	SHL determines the seedling's center of gravity. An overly long hypocotyl results in "leggy" seedlings that are top-heavy. Such seedlings are highly susceptible to lodging or stem breakage during mechanical vibration and transportation, making them unsuitable for automated transplantation.
4		Canopy spread of seedlings	SCP	SCP affects the spacing and independence of seedlings within the plug tray. An excessively large canopy spread causes the leaves of adjacent seedlings to entangle. This seriously interferes with machine vision recognition and accurate single-plant grasping by the manipulator, often resulting in leaf damage or the simultaneous grasping of multiple plants.
5		Stem uprightness of seedlings	SSU	SSU posture determines the standard grasping path of the robotic arm. Upright-growing seedlings provide an ideal vertical downward grasping trajectory. In contrast, inclined or prostrate plants significantly increase positioning complexity and the grasping error rate.
6		Stem toughness of seedlings	SST	SST determines the seedling's ability to survive non-rigid clamping or accidental bending. A stem with high toughness can tolerate elastic deformation during mechanical operations without breaking, thereby improving operational fault tolerance and seedling adaptability.
7		Seedlings stem hardness of seedlings	SSH	SSH determines its tolerance to direct clamping forces. High stem hardness prevents permanent indentation or tissue damage caused by mechanical clamps, which is crucial for ensuring healthy post-transplantation growth and preventing disease infection.

8		Substrate disintegration rate of seedlings	SSDR	SSDR is a prerequisite for the rapid establishment of the root system after transplantation. In varieties with a low root ball disintegration rate, the root ball remains intact and resists scattering when extracted from the plug tray by the manipulator. This ensures root system integrity and a high post-transplantation survival rate. Conversely, a high disintegration rate leads to the separation of roots and substrate, significantly reducing the survival rate.
9	Harvesting stage	Plant height	PH	PH determines the overall design size and operating range of the harvesting machinery. Uniform plant height within a population facilitates one-time, unified calibration of the machine, enabling efficient and continuous operation. Plants that are too tall or too short necessitate frequent mechanical adjustments, thereby reducing operational efficiency. Thus, plant height is a key agronomic trait for evaluating the suitability of peppers for mechanized harvesting.
10		Plant stem diameter	PSD	PSD reflects the mechanical strength of the plant's primary load-bearing structure. A robust main stem provides the mechanical foundation required to withstand the continuous vibrational forces generated by the harvester without lodging or snapping. This is crucial for ensuring a smooth harvesting process.
11		Plant canopy spread	PCS	PCS directly affects planting density and machinery clearance between rows. A compact plant architecture (narrow canopy spread) accommodates higher planting densities and provides sufficient inter-row space for the harvester, preventing damage and blockages caused by entangled foliage. Conversely, an excessively wide canopy creates dense inter-row spaces, restricting machine access and increasing the risk of damage to adjacent plants.
12		First bifurcation height of plant	FFH	FFH provides unobstructed operational clearance for the chassis and fruit-collecting devices of the harvester. A higher first branch effectively prevents the machinery from scraping or colliding with lower branches and leaves. For vibrating harvesters, this clearance also provides an optimal clamping position.
13		Plant lodging resistance	PLR	PLR is a prerequisite for mechanized harvesting. Lodged plants prevent the machinery from effectively grasping or vibrating the stem, resulting in high rates of unharvested fruit and significantly limiting field operational capacity and efficiency.
14		Fruiting branch toughness	FBT	FBT directly influence the rate of branch breakage during harvest. Branches with high toughness undergo elastic deformation rather than brittle fracture when subjected to vibrational or brushing forces. This significantly reduces branch and leaf impurities in the harvested product, thereby improving the purity of the commercial yield.

15	Fruit morphologic-al uniformity	FMU	FMU dictates adaptability to post-harvest processing equipment. Consistent and uniform fruit shapes facilitate the design of standardized sorting, orienting, cleaning, and destemming mechanisms, thereby enhancing the efficiency and precision of automated processing lines. Consequently, morphological indicators like fruit length and width are essential for comprehensively evaluating suitability for mechanized harvesting.
16	Fruit size uniformity	FSU	FSU reflects the developmental synchrony of the fruit. A highly uniform fruit population indicates consistent mechanical properties and maturity levels. This uniformity not only facilitates post-harvest mechanical grading but also serves as the biological foundation for achieving efficient, one-time harvesting and a high marketable yield.
17	Lowest fruit height from the ground	FGH	FGH determines the minimum working height of the harvester's fruit-collecting device. Sufficient ground clearance prevents low-hanging fruits from being crushed or contaminated with soil during machine passage, thereby minimizing harvest losses and impurity rates
18	Fruit setting position	FSP	FSP affects the contact probability with mechanical components. A concentration of fruits in the middle and outer canopy at a consistent height facilitates effective contact by mechanical brushing, vibrating, or picking devices. This minimizes unharvested fruit and maximizes harvesting efficiency.
19	Fruit color	FC	FC is closely correlated with the quality of processed peppers. Generally, a deeper, brighter red color indicates a higher quality level in the final processed product.
20	Fruit hardness	FH	FH directly dictates resistance to physical damage. Firmer fruits are less susceptible to mechanical injury during vibration-induced detachment, collision, and transportation. Therefore, firmness is a critical quality indicator for minimizing losses from the field to the processing line.
21	Fruit pedicel separation force	FSF	FSF is the critical factor for achieving effective vibration-induced detachment. A moderate separation force enables efficient fruit detachment under appropriate vibrational frequencies. If the required force is too low, pre-harvest fruit drop may occur. Conversely, an excessively high detachment force necessitates intense vibration, which can cause plant damage, branch breakage, or the harvesting of fruits with intact pedicels.

Table S3 Principal component analysis of evaluation indicators for suitability of chili peppers to mechanized transplanting and harvesting

Evaluation indicators	Principal component							
	1	2	3	4	5	6	7	8
SPH	0.301	0.696	0.333	0.211	-0.121	-0.146	0.217	0.159
SSD	0.228	0.678	0.229	-0.091	-0.369	0.114	-0.102	0.153
SHL	0.251	-0.192	0.617	0.360	0.235	-0.211	0.037	-0.004
SCP	0.438	0.406	0.629	-0.079	-0.137	0.195	0.064	0.192
SSU	-0.201	0.228	0.215	0.580	-0.404	0.142	-0.006	-0.270
SST	-0.153	0.515	-0.356	-0.099	0.328	-0.403	-0.075	0.247
SSH	-0.056	0.564	-0.343	-0.178	0.091	-0.323	0.338	0.051
SSDR	0.252	-0.325	0.427	-0.206	0.361	0.228	-0.072	0.537
PH	-0.600	0.219	-0.194	0.396	0.212	0.454	0.108	0.186
PSD	0.392	0.326	-0.322	-0.050	0.019	0.436	-0.285	0.019
PCS	0.082	0.328	-0.596	0.446	0.027	0.290	-0.049	0.241
FFH	-0.775	-0.003	0.264	0.084	0.300	0.137	0.218	0.081
PLR	0.652	-0.338	-0.253	0.186	-0.105	0.167	0.331	-0.112
FBT	-0.804	0.157	0.115	-0.084	-0.114	-0.051	0.135	-0.169
FMU	-0.273	-0.295	-0.085	-0.28	-0.446	0.036	0.050	0.446
FSU	0.320	0.248	0.145	-0.377	0.111	0.379	0.338	-0.292
FGH	-0.818	0.095	0.248	0.154	0.210	0.173	0.128	-0.018
FSP	0.192	-0.554	-0.214	0.177	-0.136	0.131	0.456	0.178
FC	-0.253	0.120	-0.085	-0.618	-0.132	0.154	0.415	-0.031
FH	0.546	0.187	-0.046	-0.100	0.565	0.163	0.035	-0.251
FSF	0.443	0.039	-0.050	0.395	0.068	-0.303	0.444	0.148
Eigenvalue	4.164	2.804	2.203	1.838	1.409	1.319	1.17	1.053
% of Variance	19.83	13.351	10.489	8.755	6.709	6.282	5.572	5.016
Cumulative %	19.83	33.181	43.671	52.425	59.134	65.417	70.989	76.005

Table S4 Principal component analysis of evaluation indicators for suitability of plate peppers to mechanized transplanting and harvesting

Evaluation indicators	Principal component						
	1	2	3	4	5	6	7
SPH	0.205	0.268	0.643	-0.367	0.156	0.300	0.197
SSD	0.232	0.583	0.504	0.201	-0.248	-0.035	0.197
SHL	0.638	0.100	-0.225	-0.365	0.395	-0.041	0.048
SCP	0.693	0.472	0.211	-0.141	0.036	-0.168	0.083
SSU	0.153	0.076	0.633	0.317	-0.090	-0.392	-0.088
SST	-0.690	-0.153	0.183	-0.337	0.116	0.435	-0.084
SSH	-0.669	-0.052	0.434	-0.124	0.000	0.344	-0.013
SSDR	0.547	0.272	-0.032	-0.170	0.416	0.047	-0.359
PH	0.047	-0.143	0.072	0.642	0.514	0.135	0.356
PSD	-0.772	0.331	-0.074	0.193	0.158	-0.192	-0.017
PCS	-0.464	0.078	0.051	0.186	0.599	-0.188	0.401
FFH	0.373	-0.489	0.183	0.115	0.058	0.307	0.200
PLR	0.354	-0.608	0.123	0.264	-0.253	-0.075	0.081
FBT	-0.088	0.523	-0.276	0.433	0.074	-0.148	-0.003
FMU	0.034	-0.318	-0.404	-0.436	0.334	-0.261	0.111
FSU	-0.190	-0.108	-0.240	-0.36	-0.278	-0.180	0.584
FGH	0.565	-0.528	-0.010	0.103	-0.061	0.200	0.223
FSP	-0.061	-0.554	0.295	0.120	0.241	-0.186	-0.228
FC	0.138	0.671	-0.064	-0.159	0.050	0.298	0.195
FH	0.224	0.025	-0.227	0.394	0.296	0.429	-0.187
FSF	0.064	0.253	-0.555	0.362	-0.373	0.371	0.067
Eigenvalue	3.751	2.981	2.197	1.998	1.638	1.382	1.097
% of Variance	17.862	14.198	10.46	9.515	7.799	6.583	5.223
Cumulative %	17.862	32.06	42.52	52.034	59.833	66.416	71.639

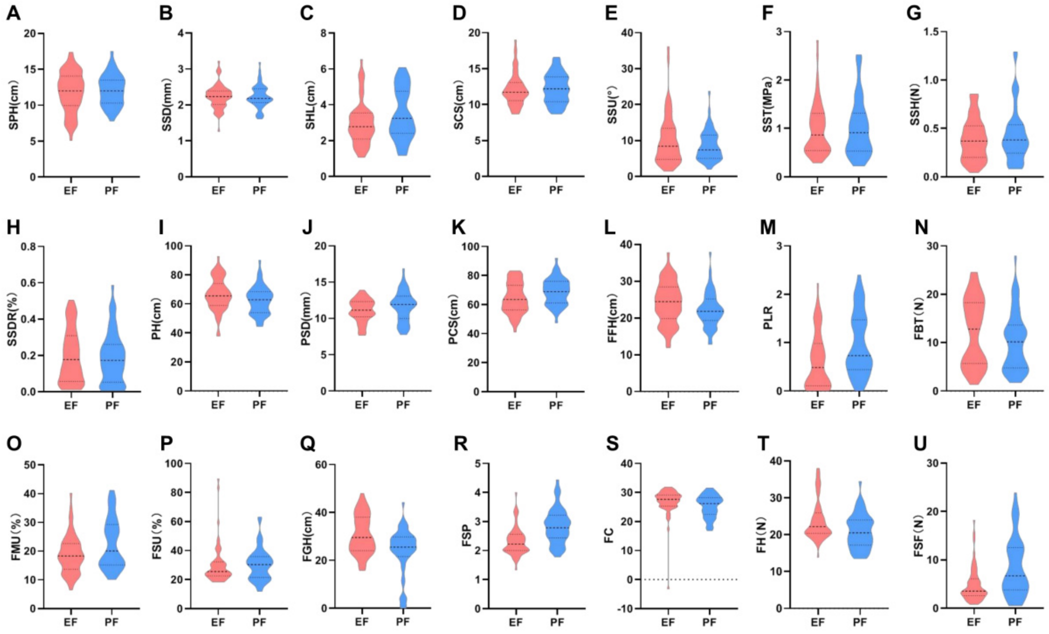

Figure S1 Variation and diversity analysis of evaluation indicators for 105 processing peppers varieties.

(A) Plant height of seedlings; (B) Stem diameter of seedlings; (C) Hypocotyl length of seedlings; (D) Canopy spread of seedlings; (E) Stem uprightness of seedlings; (F) Stem toughness of seedlings; (G) Seedlings stem hardness of seedlings; (H) Substrate disintegration rate of seedlings; (I) Plant height; (J) Plant stem diameter; (K) Plant canopy spread; (L) First bifurcation height of plant; (M) Plant lodging resistance; (N) Fruiting branch toughness; (O) Fruit morphologic-al uniformity; (P) Fruit size uniformity; (Q) Lowest fruit height from the ground; (R) Fruit setting position; (S) Fruit color; (T) Fruit hardness; (U) Fruit pedicel separation force.

EF: erect-fruit pepper group; PF: pendent-fruit pepper group

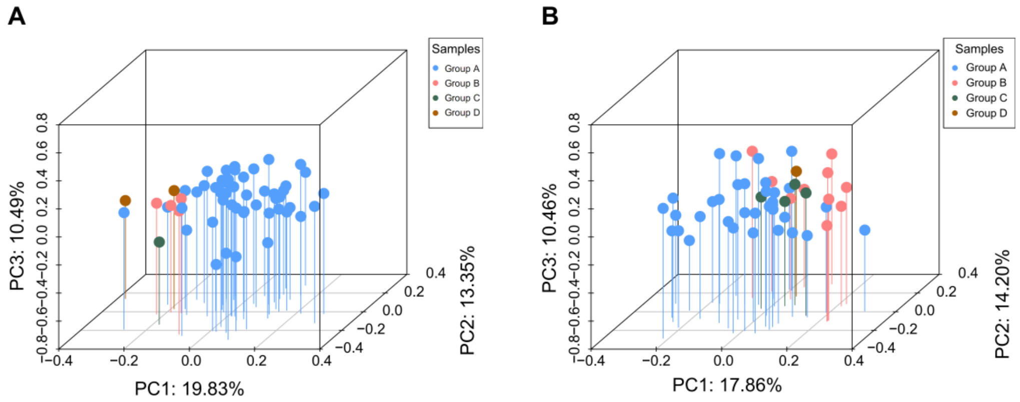

Figure S2 Score plots of principal components 1-3 based on principal component analysis

(A) erect-fruit peppers; (B) pendent-fruit peppers
